# Peer Review of “Artificial Intelligence in Healthcare: 2023 Year in Review (Preprint)”

**DOI:** 10.2196/65151

**Published:** 2024-09-19

**Authors:** Vanessa Fairhurst, Christopher Steven Marcum, Courtney Haun, Paulina Boadiwaa Mensah, Femi Qudus Arogundade, Ruchi Pathak Kaul, Sidharth Narayanan, S Shah

**Affiliations:** 1 PREreview, Oxford, United Kingdom, 44 123456789; 2 PREreview, Washington, DC, United States; 3 NASA, Huntsville, AL, United States; 4 SnooCODE, Accra, Ghana; 5 ASAPbio, Ibadan, Nigeria; 6 University College London, London, United Kingdom; 7 IGDORE, Karachi, Pakistan

**Keywords:** machine learning, artificial intelligence

*This is a peer-review report submitted for the preprint “Artificial Intelligence in Healthcare: 2023 Year in Review.”*

This review is the result of a virtual collaborative live review discussion organized and hosted by PREreview and JMIR Publications. The discussion was joined by 34 people: 2 facilitators, 3 members of the JMIR Publications team, 3 authors, and 26 live review participants. Rachel Grasfield, Ranjani Harish, Kolapo Oyebola, Arya Rahgozar, Renato Sabbatini, Nour Shaballout, and Trevor van Mierlo wished to be recognized for their participation in the live review discussion, even though they have not contributed to authoring the review below. We thank all participants who contributed to the discussion and made it possible for us to provide feedback on this preprint.

## Summary

- Research question: The review [1] aims to understand the development and applications of artificial intelligence (AI) in health care, assessing the prevalence and impact of AI methods in biomedical research. The focus is on the frequency and types of publications in 2023, aiming to provide a comprehensive overview of the current AI landscape in health care and identify areas needing further research. It also aims to address the specialties in medicine with greater use of AI.
- Research approach: The authors employed a mixed methods approach, combining classical bibliometric analysis and advanced deep learning techniques to analyze PubMed data. They established search criteria to collect papers published in 2023 related to AI and machine learning in health care, identifying subcategories and medical specialties within the dataset.
- Research findings: An increase in AI-related health care publications was observed in 2023, with a total of 23,306 articles, which constitutes a 133.7% increase from the previous year. The analysis revealed a differential uptake of AI models across medical fields, with imaging being the most prevalent. The review also noted a spread across various specialties, with cardiology, gastroenterology, ophthalmology, and general clinics being prominent, while psychiatry had fewer publications.
- Interesting aspects: The review provided insights into future AI trends in medicine and health care. The growth of image-based publications and the usefulness of AI in specific health care specialties were particularly noted. The review provides a valuable snapshot of AI’s role in health care research in 2023, highlighting the rapid growth and diverse applications of AI technologies across medical specialties. The authors found AI use in imaging was the most prevalent among the specialties and that other fields, such as gastroenterology, ophthalmology, and psychiatry are promising for future use of AI.
- Relation to published literature: The authors attempt to establish their review as a foundational reference for the current state of AI in health care literature for the past year and to set the stage for future comparative analyses. However, prior work in reviewing the use of AI in certain medical fields, such as robotics and hospital administration, which are prevalent in the established literature, have not been thoroughly addressed in the paper and could warrant greater discussion.
- Strengths and weaknesses: The review’s reproducibility and the use of mixed methods for bibliometric analysis are strengths of the paper. However, the concept of “mature” AI models could use improvement because of its vagueness, and the limited keyword search is a potential weakness. Suggestions for improvement include adhering to reporting guidelines like PRISMA-ScR (Preferred Reporting Items for Systematic Reviews and Meta-Analyses Extension for Scoping Reviews), clarifying the definition of “mature” research, and considering the inclusion of “healthcare” in the initial search criteria. Additionally, the time frame of a single recent year is very short considering the fact that AI has been around since the 1956 Dartmouth conference and has long been used in the forefront of health research. The authors should consider suggesting that future studies with longer time frames may be carried out as the applications of AI progress over the years.

Below we list major and minor concerns that were discussed by participants of the live review, and where possible, we provide suggestions on how to address those issues.

## Major Concerns

- The authors should more clearly articulate the generalizability to only within the PubMed corpus and, feasibly, within the search results of their query choice. Alternatively, the authors could consider expanding their search terms to include additional relevant keywords to be more holistic of the range of AI uses within health research or expand their database search outside of PubMed to ensure a broader inclusiveness of the literature.
- The Bidirectional Encoder Representations From Transformers (BERT)–based maturity classification model and other AI classification methods encountered difficulties in correctly categorizing publications into their medical specialties. This challenge stemmed from BERT’s vocabulary limitations, as it was only trained on a specific set of tokens, making it struggle with unfamiliar or uncommon terms not present in its training data. The observed accuracy of these models ranged from 30% to 68%, suggesting frequent misclassifications or inaccurate categorizations of publication specialties. The authors should consider further improvements and training of AI models to enhance the accuracy of specialty classification and reduce the occurrence of false positives.
- The adoption of multimodal models incorporating diverse data types remains restricted, with image data largely prevailing in AI applications within health care, despite the vast array of available data. The authors should consider implementing multimodal models that combine different data formats such as text, tabular, and voice, as this could significantly enhance the effectiveness and adaptability of AI in health care.
- Authors should consider how their study deviates from the “gold standard” PRISMA framework and guidelines and discuss why their method would be preferred over PRISMA in this specific case.
- The figures need considerable revision before this would be acceptable for publication. Reviewers recommend the following changes:
- For Figure 4B, authors should include a definition of terms to make it clear what exactly AI, machine learning, large language model, natural language processing, etc, refers to in context of the papers scoped.
- To improve readability, the authors should organize the visualizations under subheaders. For example, Figures 3 and 5 showing visualizations across health care specialties can be grouped together under one subheader.
- For Figure 3, imaging should be analyzed separately as it is a major outlier. For example, breaking it down into the specialties in which imaging was applied, as imaging cuts across all specialties (eg, radiology, oncology, histology)
- Data and code should be made freely accessible by default and not incumbent on the reader to “request the data” from the authors. The authors should remedy this oversight before submitting for publication.

## Minor Concerns

- The authors should more carefully define “maturity.” Typically, in AI, maturity refers to a property of how well established the use of AI is within an organization. It is not clear from the paper how that generalizes to a medical field per se.
- The paper should lavish more details on the geographic subanalysis and results.
- The authors should provide more details on the justification of why they chose the specific search terms used to define the sampling frame. If possible, the authors should point to an empirical determination of why those specific terms were chosen over other reasonably good choices.
- Authors should consider providing more details about the papers scoped, for example, distribution across journals/frequency of journals and most common author-provided keywords.
- The authors should explain the term “infodemic” as used in the introduction perhaps by referencing the World Health Organization definition.
- Authors should provide support for their claims on the “exponential growth of AI,” for example, by referencing past studies or including visualizations to illustrate this.

## Concluding Remarks

The reviewers agree that this paper makes an important and timely contribution to the literature. There are several issues that should be addressed prior to finalization of the manuscript—however, the reviewers believe these concerns are easily addressable by the authors.

## Abbreviations

AI: artificial intelligence
BERT: Bidirectional Encoder Representations From Transformers
PRISMA-ScR: Preferred Reporting Items for Systematic Reviews and Meta-Analyses Extension for Scoping Reviews
